# Investigating β-*N*-Methylamino-l-alanine Misincorporation in Human Cell Cultures: A Comparative Study with Known Amino Acid Analogues

**DOI:** 10.3390/toxins9120400

**Published:** 2017-12-14

**Authors:** Rianita van Onselen, Simoné Downing, Gabré Kemp, Tim Downing

**Affiliations:** 1 Department of Biochemistry and Microbiology, Nelson Mandela University, P.O. Box 77000, Port Elizabeth 6031, Africa; Simone.Downing@mandela.ac.za; 2 Department of Microbial, Biochemical and Food Biotechnology, University of the Free State, P.O. Box 339, Bloemfontein 9300, Africa; kempg@ufs.ac.za

**Keywords:** β-*N*-methylamino-l-alanine, BMAA, misincorporation, analogues, m-tyrosine, l-4-fluorophenylalanine

## Abstract

Misincorporation of β-*N*-methylamino-l-alanine (BMAA) into proteins has been proposed to be a mechanism of toxicity to explain the role of BMAA in neurodegenerative disease development. However, studies have shown that all detectable BMAA can be removed from proteins by SDS-PAGE purification and that the toxicity of l-canavanine cannot be reproduced in prokaryotes or in a rat pheochromocytoma cell line, strongly indicating that the misincorporation hypothesis of BMAA should be re-investigated. The aim of this study was therefore to determine if BMAA misincorporates into proteins in cells of human origin with subsequent misincorporation-type toxicity. Almost complete loss of viability in response to exposure to l-4-fluorophenylalanine and l-m-tyrosine was observed in all of the cell lines, corresponding to a concentration-dependent increase of the analogues in protein extracts from exposed cells. In contrast, BMAA exposure resulted in slight toxicity in one of the cell lines but the observed toxicity was not the result of misincorporation of BMAA into proteins, as no BMAA was detected in any of the SDS-PAGE purified protein extracts that were obtained from the cells following BMAA exposure. The results show that BMAA is not misincorporated into human proteins and that misincorporation is not a valid mechanism of toxicity.

## 1. Introduction

Exposure to β-*N*-methylamino-l-alanine (BMAA), a non-encoded amino acid produced by cyanobacteria [1] and diatoms [2], has been linked to the development of neurodegenerative diseases such as Amyotrophic Lateral Sclerosis/Parkinsonism Dementia complex (ALS/PDC) and Alzheimer’s disease [3,4,5,6,7,8]. The following mechanisms of BMAA toxicity have been proposed: excitotoxicity of the β-*N*-carboxy BMAA adduct that forms spontaneously in the presence of bicarbonate [9,10,11,12], the formation of toxic metabolites such as formaldehyde (capable of cross-linking proteins and nucleic acids), 2,3-diaminopropionic acid,z and methylamine upon metabolism of BMAA [13,14,15], and misincorporation of BMAA into proteins in place of l-serine [16]. While there is substantial evidence to support the production of toxic metabolites and the excitotoxic nature of BMAA [11,17,18,19,20,21,22,23,24,25,26,27] (albeit relatively weak excitotoxicity [28,29] comparable to the dietary supplement β-alanine [30]), misincorporation of BMAA into proteins as a mechanism of toxicity has yet to be confirmed in a natural system.

Murch et al. [31] established that most of the BMAA found in cycad flour and fruit bat tissue is associated with proteins rather than being part of the free amino acid pool, as previously thought, and that BMAA could only be removed from proteins by overnight acid hydrolysis at 110 °C, prompting them to suggest misincorporation as a mechanism of toxicity. In support of this hypothesis, Dunlop et al. [16] reported a reduced concentration of protein-associated BMAA and decreased toxicity in the presence of excess l-serine in SH-SY5Y neuroblastoma cells, which they attributed to competition for protein incorporation. Similarly, Glover et al. [32] reported BMAA–protein associations in proteins produced in a cell-free *Escherichia coli* in vitro expression system, which they also attributed to misincorporation since BMAA could not be removed by dithiothreitol (DTT) and sodium dodecyl sulfate (SDS) washing. Interestingly, based on the observed association of the BMAA isomers *N*-(2-aminoethyl)glycine and 2,4-diaminobutyric acid with proteins, Glover et al. [32] suggested that these isomers also misincorporate into proteins, although they do not suggest how this might occur. However, the inability to remove BMAA by DTT and SDS washing does not mean that BMAA is part of the primary structure of the proteins, since additional purification of proteins using sodium dodecyl sulphate-polyacrylamide gel electrophoresis (SDS-PAGE) or immobilized metal ion affinity chromatography has been shown to remove all detectable amounts of BMAA from tested proteins that previously yielded BMAA only after acid hydrolysis [33,34].

Misincorporation of non-encoded amino acids into proteins in place of standard encoded amino acids is a phenomenon that has been investigated since the 1950s [35,36,37,38]. Naturally occurring amino acid analogues are commonly found in leguminous plants, where they function as nitrogen storage compounds, allelopathic agents, and/or grazing deterrents (reviewed in [39,40]). l-canavanine is one of the most widely studied natural amino acid analogues that misincorporates in place of l-arginine in proteins [41,42]. Ingestion of large quantities of l-canavanine has been linked to the development of autoimmune disorders such as systemic lupus erythematosus (SLE) in humans and non-human primates [43,44,45,46], and to growth retardation in chicks [47]. The replacement of the δ-methylene group in arginine with an oxygen in l-canavanine, results in a decrease in the p*K*_a_ of arginine from 12.48, to 7.01 in l-canavanine [48]. As a result of this reduced basicity that renders l-canavanine slightly more reactive, the overall charge status of the side chain group is altered. Therefore, if l-canavanine is randomly misincorporated, the proteins are likely to have an altered three-dimensional structure and these altered proteins are recognized as foreign by a host immune system. Consequently, a host immune response is triggered, explaining the typical symptoms associated with SLE: the production of antibodies against red blood cells that results in anemia, lowered complement components in serum, the presence of antibodies to double-stranded DNA (possibly as a result of misincorporation into histones) and nuclear antigens, and the accumulation of immunoglobulin and complement components in the skin and kidneys [44].

The misincorporation of l-azetidine-2-carboxylic acid (Aze), a natural amino acid first isolated from *Convallaria majalis* [49], into proteins in the place of proline has also been extensively studied in a number of cell and animal models [50,51]. Proline-rich proteins such as collagen, keratin and hemoglobin are most susceptible to misfolding when Aze is present during de novo protein synthesis (reviewed in [39]). Consequently, early in utero exposure to Aze in a number of rodent models results in malformation of the lungs, inner ear, hair, vertebra, teeth, long bones, neural crest, and other structures [52,53,54]. Despite numerous animal studies and the discovery of Aze in commonly consumed garden beets (*Beta vulgaris*) [51], the role of Aze in the development of human diseases has remained relatively unexplored. However, an interesting hypothesis was raised regarding the possible contribution of Aze in the development of multiple sclerosis (MS) in humans [55]. This hypothesis was based on an unusual incidence of enzootic ataxia (also known as swayback) in at least 60% of newborn lambs in a single flock and in a single breeding season in Alberta, Canada in 1972 [56]. The specific flock was maintained on the same farm without any incidence of swayback for 20 years. The only modification that was introduced in that specific breeding season was the addition of sugar beet-top silage, known to contain Aze, as the main source of roughage during pregnancy, lambing, and early lactation [55,56]. The symptoms of swayback include ataxia, head shaking, physical weakness, trembling, incoordination, and swaying of the hind quarters, concurrent with widespread pathological demyelination that resembles the pathological lesions observed in MS. Rubenstein therefore hypothesized that misincorporation of Aze into the myelin basic protein, specifically in the highly conserved hexapeptide sequence PRTPPP, can lead to a disruption in myelination and subsequently cause MS in humans and swayback in animals. This hypothesis is further supported by the proposed link between MS prevalence and the geographical distribution of sugar beet agriculture, specifically in Hokkaido (Japan), Finland, the Middle East, Sardinia, Alberta (Canada), and the Orkney Islands [55].

The unusual ability of fine-leaf fescue grasses (*Festuca* spp.) to outcompete other plants has been attributed to their ability to synthesize and excrete l-m-tyrosine (m-tyr), another non-encoded amino acid, into the rhizosphere [57]. The toxicity of m-tyr has been proposed to be the result of misincorporation of m-tyr into proteins in the place of l-phenylalanine and this has been reported to occur in prokaryotes [58,59], mammalian cell culture [60], and plants [57], with consequent growth retardation and decreased viability. It has been reported that m-tyr and other oxidized byproducts of free l-phenylalanine can be formed in the presence of hydroxyl radical species that often accumulate under conditions of cellular stress [61,62], leading to the misincorporation of m-tyr during de novo protein synthesis [63]. The role of these cytotoxic mistranslations in human and animal disease development remains unclear.

The toxicity observed in animals exposed to BMAA differs greatly from the toxicity associated with exposure to known amino acid analogues such as l-canavanine and Aze as described above. The replacement of l-serine by BMAA at any significant level, would have widespread and severe implications for the organism given the crucial role of l-serine in many proteins. l-Serine plays a key catalytic role in many enzymes and in hydrogen bonding within proteins, and can undergo glycosylation and its hydroxyl is a site for protein phosphorylation. The importance of l-serine in all of these critical metabolic aspects makes it unlikely that the toxicity would be limited to the central nervous system unless BMAA specifically and rapidly accumulates in these target tissues. There is some evidence to suggest that this does happen [64], but there is also evidence of liver, kidney, and muscle accumulation after intravenous administration, with less than 0.08% of the original dose being the peak concentration in the brain at two hours, an amount comparable to that seen in other tissues [65]. Similarly, fairly wide tissue distribution of BMAA in fruit bats has been reported, with much of the BMAA being in the skin and fur [66]. In non-albino mice, an accumulation of intravenously administered BMAA was noted in the eye and hair follicles and in all tissues with high cell turnover such as salivary glands, bone marrow and gastrointestinal mucosa [67]. In *Rana temporaria* subcutaneously administered BMAA accumulated in all pigmented tissues including eye, liver, melanocytes surrounding blood vessels and visceral organs, as well as pigmented neurons and meninges [67]. Given this distribution, and similar half-life values in the different tissues [65], toxic effects would be expected in all tissues containing BMAA if misincorporation occurred in place of the important l-serine moiety. Furthermore, BMAA is described as a late onset toxin with symptoms evident only long after exposure [14,31,68]. However, both free amino acid and in the protein associated BMAA has been reported to be cleared quickly from all tissues of rats that were exposed to BMAA [15,65], making late-onset misincorporation toxicity highly unlikely. In contrast, the onset of gross toxicological features of misincorporation follows quickly after ingestion of amino acid analogues. These clear toxicological differences between BMAA and known amino acid analogues, suggest that misincorporation of BMAA may not occur in animals. Additionally, the reduction in growth rate caused by misincorporating amino acid analogues in bacteria, also did not occur in bacteria exposed to BMAA [34]. That the known amino acid analogues generally misincorporate in both eukaryotic and prokaryotic examples would make BMAA unique in this regard and require some specific differences in the seryl-tRNA synthetases for this to be the case. Nonetheless, the absence of analogue toxicity type symptoms on exposure to BMAA, and the absence of misincorporation in prokaryotes, along with the ability to remove BMAA from proteins by SDS-PAGE, suggest that BMAA may not be misincorporated into proteins, as has been hypothesiszd.

The absence of reports of BMAA toxicity in cell cultures other than neuronal cells (e.g., primary human neurons [26], rat olfactory unsheathing cells [27], human neuroblastoma SH-SY5Y [17,33]) further challenges the misincorporation hypothesis. Although Dunlop et al. [16] reported the misincorporation of BMAA in a human lung fibroblast cell line and in human umbilical vein endothelial cells, by virtue of detection of BMAA in the protein fraction, no toxicity in these cell lines was reported whereas toxicity was reported for the neuroblastoma cell line used in the same study, suggesting excitotoxicity as a mechanism. Van Onselen et al. [69] showed that differentiation of a rat pheochromocytoma cell line (PC12) with nerve growth factor, with resulting expression of glutamate receptors, was required in order to achieve BMAA toxicity, presumably via an excitotoxic mechanism. Differentiation of the same cell line was not required to observe toxicity in response to l-canavanine exposure, indicating different mechanisms of toxicity of these two compounds.

In his review published in 1962, Richmond [70] stipulated two general and fundamental requirements for a non-encoded amino acid to be considered an amino acid analogue that can be misincorporated: Firstly, if the side chain group is ionizable, it must produce the same type of ion as the side chain of the amino acid that is being replaced or it must be uncharged at physiological pH. Due to this requirement, there are only a few common side chain substitutions that permit non-encoded amino acids to be successful amino acid analogues. In terms of ionization state, these substitutions include -F for -H, an -O- or -S- for -CH_2_-, or -NH_2_ for -OH. Secondly, the analogue must be of a similar shape and size to the amino acid that is being replaced. An increase in side chain length by -CH_2_- is a feature of some of the larger amino acid analogues such as ethionine for methionine (reviewed in [70]). Consequently, natural amino acid analogues that differ in size from their protein counterpart have only been identified for the larger amino acids such as l-arginine, l-phenylalanine, l-tyrosine, l-tryptophan, l-methionine and l-leucine since subtle side chain substitutions are relatively small compared to the large overall size of these amino acids. No natural amino acid analogues have been identified for the smaller amino acids such as l-alanine, l-glycine, l-serine and l-threonine, as a substitution in the side chains of these amino acids will result in a relatively large change in the overall size and shape of these amino acids.

**Figure 1:**
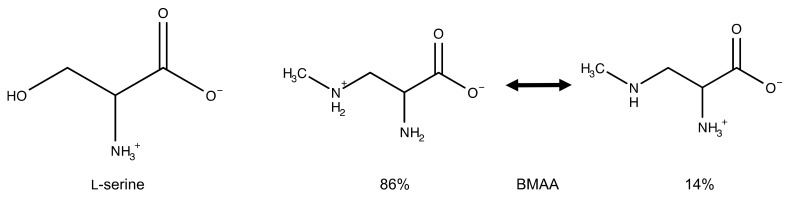
Illustrating the structural differences between l-serine and BMAA at physiological pH.

Based on the structures of BMAA and l-serine at physiological pH (Figure 1), BMAA does not fit the requirements stipulated by Richmond [70] for non-encoded amino acids to be considered successful amino acid analogues. BMAA has an extra methyl group and an exchange of the side chain hydroxyl for an amine. In addition, 86% of the secondary amine of BMAA is ionized at physiological pH [71], while the side chain of l-serine is uncharged. The specificity of seryl-tRNA synthetases is mainly determined by two factors: the interaction of the side chain hydroxyl group with Thr-380 in motif 3 of the enzyme and by the small size of the side-chain binding pocket [72]. Therefore, the exchange of the hydroxyl group in l-serine for the less reactive amino group in BMAA, and the increase in size by a methyl group, make the successful charging of tRNA^ser^ with BMAA, unlikely.

The hypothesized misincorporation of BMAA, although widely mentioned as a mechanism of toxicity, thus lacks animal toxicity, in vitro, and biochemical evidence. Furthermore, the observation that BMAA in the central nervous system is predominantly in the d-form [73], makes misincorporation as a mechanism of central nervous neurotoxicity highly unlikely [74]. Evidence for non-primary protein association in favor of misincorporation also exists [34]. Furthermore, despite reports that BMAA does not misincorporate into proteins in bacteria or in a rat pheochromocytoma cell line [34,69] it has been suggested that the lack of prokaryote misincorporation may not hold true in eukaryotes [75], although the authors cite Glover et al. [32] in support of their hypothesis, despite their use of a prokaryotic expression system. In this study, human cell lines were therefore used to evaluate BMAA misincorporation using both toxicological markers and amino acid compositional analysis of purified proteins from exposed cell lines, with reference to known misincorporating amino acid analogues.

## 2. Results and Discussion

The toxicity of BMAA in comparison to l-4-fluorophenylalanine (FPA) and m-tyr was determined by evaluating metabolic activity and apoptosis/necrosis in three human cell lines, so as to determine whether BMAA produced the same type of toxicity at similar concentrations as the known misincorporating amino acid analogues. The rationale was that if BMAA did not produce any toxicity, or produced a different profile of apoptosis and necrosis with increasing concentrations in any of the cell lines, the mechanism of toxicity must differ. Amino acid ratios, including the ratio of the analogue to the replaced encoded amino acid, of SDS-PAGE purified proteins from exposed cell lines were then used to determine whether any toxicity observed was the result of misincorporation of the analogue.

### 2.1. Metabolic Activity

**Figure 2:**
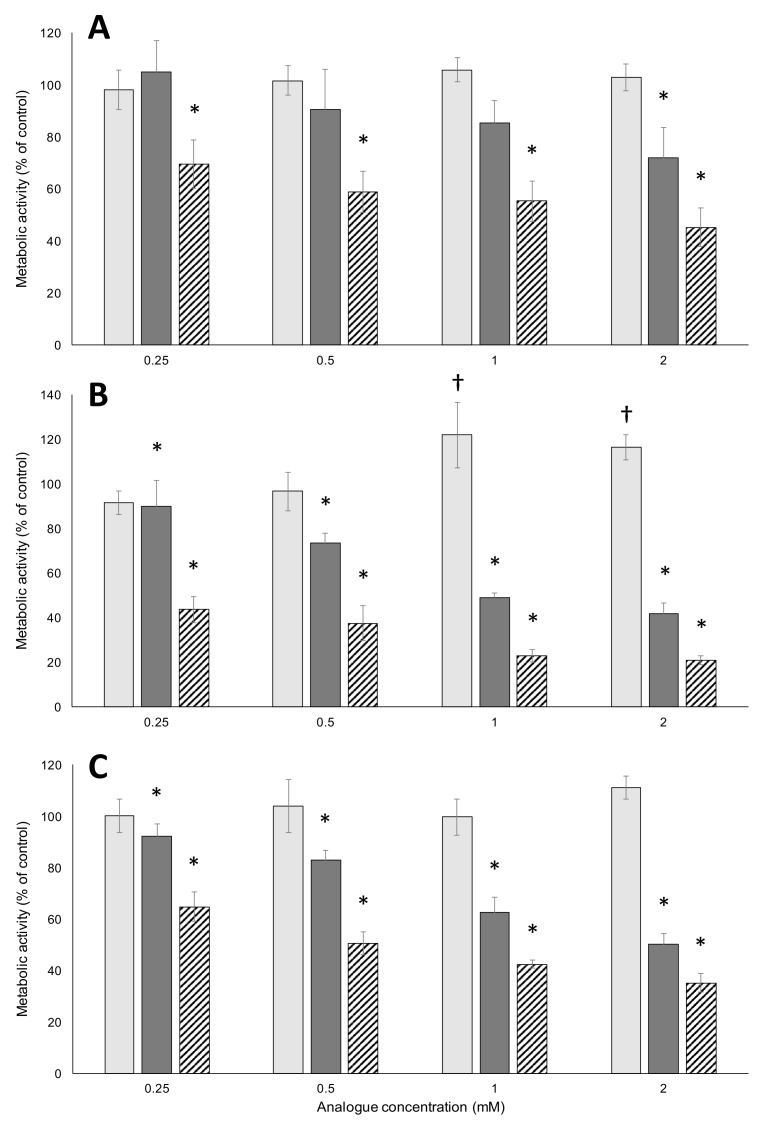
Metabolic activity of cells exposed to amino acids at various concentrations. HepG2 (**A**), Caco-2 (**B**) and HeLa (**C**) cells were exposed to BMAA (light gray bars), l-m-tyrosine (dark gray bars) and l-4-fluorophenylalanine (hatched bars) over a range of concentrations, indicated on the x-axis, for 48 h. Metabolic activity was subsequently assayed using the MTT assay. The error bars indicate ± standard deviation from the mean of six replicate samples (*n* = 6). An asterisk (*) indicates a significant decrease from vehicle-treated controls (*p* ≤ 0.05), while a dagger (†) indicates a significant increase from vehicle-treated controls (*p* ≤ 0.05).

Figure 2 shows the activity, as measured by the metabolic reduction of 3-(4,5-dimethylthiazol-2-yl)-2,5-diphenyltetrazolium bromide (MTT), of FPA, m-tyr, and BMAA-exposed HepG2 (A), Caco-2 (B) and HeLa (C) cells at analogue concentrations ranging from 0.25 mM to 2 mM. FPA decreased metabolic activity at all the concentrations tested in all cell lines. m-Tyr decreased metabolic activity in both Caco-2 and HeLa cell lines at all tested concentrations, but only at the highest tested concentration in HepG2 cells. BMAA had no significant effect on HepG2 or HeLa cells at any of the tested concentrations. Interestingly, in Caco-2 cells, BMAA at 1 mM and 2 mM significantly enhanced the metabolic activity. These data indicate an absence of BMAA toxicity in these non-neuronal cell lines.

### 2.2. Apoptosis/Necrosis

**Figure 3:**
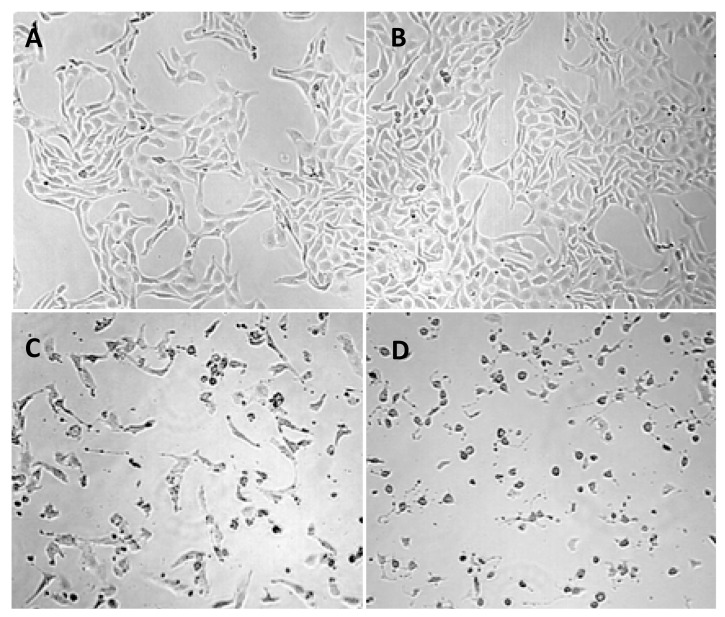
Representative images of Caco-2 cells following 48 h of exposure to the various amino acids. (**A**) depicts the overall shape and size of untreated control Caco-2 cells; (**B**) shows the same cells exposed to BMAA for 48 h; (**C**) is a representative image of Caco-2 cells exposed to m-tyr; while (**D**) shows the same cells exposed to FPA. The images are representative of at least nine fields of views taken for six replicate samples.

The FPA and m-tyr toxicity in Caco-2 cells after 48 h of exposure to 2 mM of each of the amino acids is clearly visible in Figure 3C,D. Cells appear shrunken and numbers are greatly reduced. In contrast, the BMAA treated cells (Figure 3B) at the same exposure concentration (2 mM) appear healthy without obvious signs of cellular stress.

**Figure 4:**
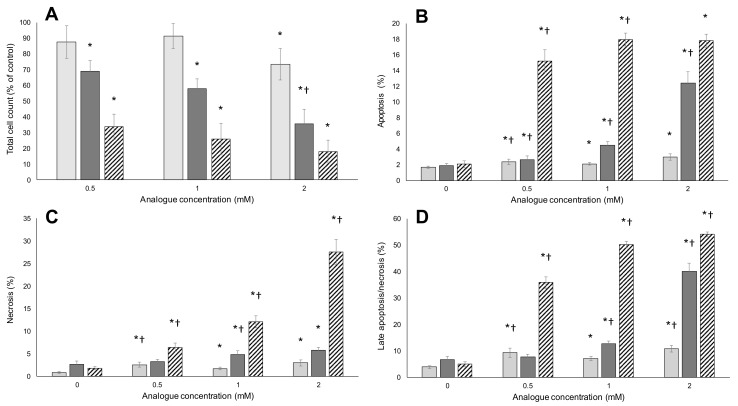
Total cell counts and percentage apoptotic and necrotic HepG2 cells measured after exposure to the respective amino acids. Following 48 h of exposure to varying concentrations of BMAA (light gray bars), m-tyr (dark gray bars) and FPA (hatched bars), total cell counts (**A**) were determined; as well as the percentage of apoptotic (**B**); necrotic (**C**) and late apoptotic/necrotic (**D**) cells. The averages of six replicate samples were plotted (*n* = 6) and the error bars denote ± standard deviation from the mean. Asterisks (*) indicate significant (*p* ≤ 0.05) differences from vehicle-treated controls. A dagger (†) indicates a significant difference from the previous (lower) concentration (*p* ≤ 0.05).

Following 48 h of treatment with BMAA, m-tyr or FPA at various concentrations, total cell counts, apoptosis, necrosis and late apoptosis/necrosis were measured for HepG2 (Figure 4) and Caco-2 (Figure 4) cells using a combination of Hoechst nuclear staining and Annexin V-FITC staining. 

**Figure 5:**
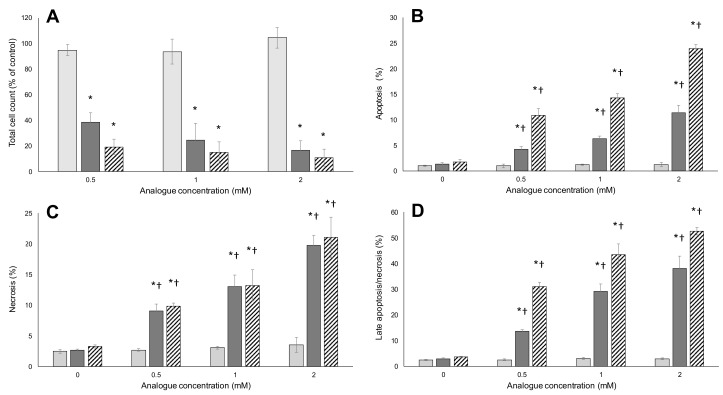
Total cell counts and percentage apoptotic and necrotic Caco-2 cells measured after exposure to the respective amino acids. Following 48 h of exposure to varying concentrations of BMAA (light gray bars), m-tyr (dark gray bars) and FPA (hatched bars), total cell counts (**A**) were determined; as well as the percentage of apoptotic (**B**); necrotic (**C**) and late apoptotic/necrotic (**D**) cells. The averages of six replicate samples were plotted (*n* = 6) and the error bars denote ± standard deviation from the mean. Asterisks (*) indicate significant differences from vehicle-treated controls. A dagger (†) indicates a significant difference from the previous (lower) concentration (*p* ≤ 0.05).

A concentration-dependent decrease in total cell numbers was observed for both HepG2 (Figure 4A) and Caco-2 (Figure 4A) cells exposed to m-tyr and FPA. A slight but significant decrease in cell number was observed for HepG2 cells exposed to BMAA at the highest tested concentrations (2 mM) but BMAA did not affect cell growth and division at any of the tested concentrations in Caco-2 cells (Figure 5). The different response of cells to BMAA, compared to known misincorporating amino acid analogues, suggests that BMAA, where toxic at the highest concentration for the one cell line, might have a different mechanism of toxicity. This is supported by the absence of an effect by BMAA on MTT reduction.

**Figure 6:**
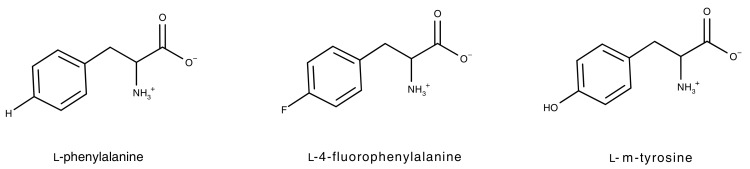
Illustrating the structural difference between l-phenylalanine and the two amino acid analogues, l-4-fluorophenylalanine and l-m-tyrosine.

FPA resulted in almost complete loss of viability when the percentages of apoptotic (Figure 4B and Figure 5B), necrotic (Figure 4C and Figure 5C) and late apoptotic/necrotic (Figure 4D and Figure 5D) are summed in both HepG2 and Caco-2 cells, with the greatest percentage of FPA-treated cells in the late apoptotic/necrotic phase. Similarly, HepG2 and Caco-2 cells were greatly affected by exposure to m-tyr, but to a lesser extent than what was observed for FPA treatment due to the lesser charge/structure similarity to the encoded amino acid and consequent lower misincorporation rate (shown in Figure 6). The replacement of an aromatic hydrogen with a fluorine results in molecules with similar characteristics, as explained by Richmond [70]. Although the para-fluorine atom in FPA is larger than the para-hydrogen atom in phenylalanine, the aromatic C-H and C-F bond lengths are very similar, the fluorine in this position is fairly unreactive, it has electronic characteristics similar to hydrogen, and it has negligible effects on the resonance of the aromatic ring. Replacement of the para-hydrogen with a hydroxyl group results in a longer C-OH bond length compared to C-H or C-F, and slightly altered chemical properties, making m-tyr misincorporation through successful charging of tRNA^Phe^, less frequent. The greatest percentage of the m-tyr exposed cells were in the late apoptotic/necrotic phase, as was seen for FPA treatment. Interestingly, BMAA induced slight but significant levels of apoptosis/necrosis in HepG2 cells (Figure 4B–D) although there was no increase in apoptosis or necrosis with increasing BMAA concentration, suggesting that the mechanism of BMAA toxicity was not a function of misincorporation. Furthermore, no toxicity was observed for BMAA-exposed Caco-2 cells in any of the measured parameters (Figure 5). Misincorporation-based toxicity, although potentially variable between cell lines for different analogues, must occur in all cell lines.

### 2.3. HPLC/MS-MS Analysis of Amino Acids

For confirmation of misincorporation, the ratio of analogue to replaced encoded amino acid must increase with increasing misincorporation, which must increase for increasing concentration of the analogue in the culture medium [60]. The ratio of the replaced amino acid, relative to other encoded amino acids in proteins should also decrease with increasing exposure to misincorporating analogues. The data presented here are for SDS-PAGE purified protein extracts (required to remove surface associated BMAA [30]) from the different cell lines exposed to FPA, m-tyr, or BMAA.

#### 2.3.1. l-m-Tyrosine Analysis

**Figure 7:**
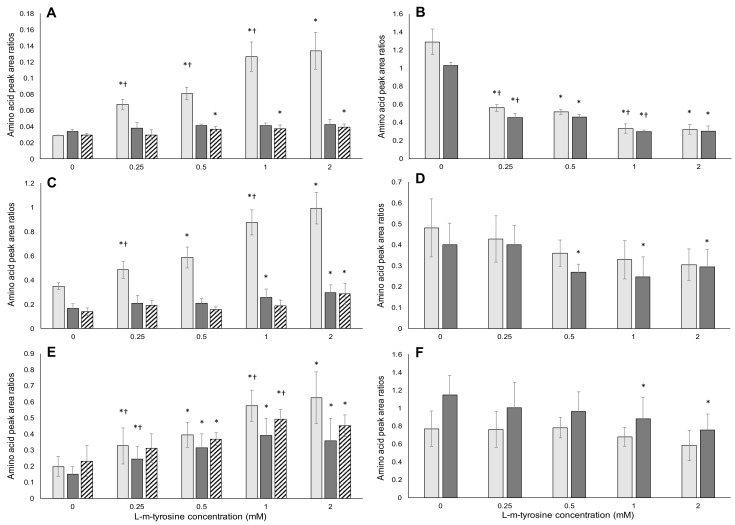
Amino acid analysis of cells exposed to l-m-tyrosine. The left-hand plots (**A**,**C**,**E**) show the ratios of m-tyr to the replaced l-phenylalanine, and two other amino acids. The right-hand plots (**B**,**D**,**F**) show the ratios of the replaced l-phenylalanine to other encoded amino acids. The top row of plots (**A**,**B**) is for HeLa cells; the middle row (**C**,**D**) for HepG2 cells; and the bottom row (**E**,**F**) for Caco-2 cells. The light gray bars in panels (**A**,**C**,**E**) represent the amino acid peak area ratios of l-m-tyrosine/l-phenylalanine. The dark gray bars in (**A**,**C**,**E**) show the m-tyr/l-serine ratios. In A, the hatched bars indicate the m-tyr/l-glycine ratios, whereas in C and E the hatched bars indicate 100 × m-tyr/l-glycine ratios. The light gray bars in (**B**,**D**,**F**) indicate the l-phenylalanine/l-serine ratios. In (**B**), the dark gray bars indicate the l-phenylalanine/l-glycine ratios, while in (**D**,**F**), the dark gray bars indicate 100 × l-phenylalanine/l-glycine ratios. The averages of five replicate samples were plotted (*n* = 5) and error bars denote ± standard deviation from the mean. An asterisk (*) indicates a significant difference from control samples (*p* ≤ 0.05) and a dagger (†) indicates a significant difference from the previous (lower) concentration (*p* ≤ 0.05).

HPLC/MS-MS analysis of the hydrolyzed gel-purified proteins extracted from the cells exposed to m-tyr (Figure 7), shows an exposure-concentration dependent increase in the ratio of m-tyr/l-phenylalanine in all of the exposed cell lines. This represents the increasing replacement of l-phenylalanine with m-tyr with increasing m-tyr exposure concentration. There is also a clear trend of increasing m-tyr/l-serine and m-tyr/l-glycine ratios with increasing m-tyr exposure concentration in all of the cell lines. Similarly, there is a decrease in the ratios of l-phenylalanine/l-serine and l-phenylalanine/l-glycine, as more of the l-phenylalanine is replaced with increasing m-tyr exposure concentrations.

#### 2.3.2. l-4-Fluorophenylalanine Analysis

**Figure 8:**
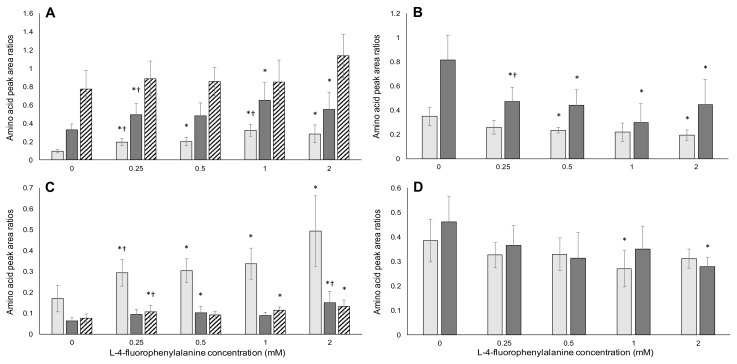
Amino acid analysis of cells exposed to l-4-fluorophenylalanine. The left-hand plots (**A**,**C**) show the ratios of FPA to the replaced encoded amino acid, and two other amino acids. The right-hand plots (**B**,**D**) show the ratios of the replaced l-phenylalanine to other encoded amino acids. The top row of plots (**A**,**B**) is for HepG2 cells, and the bottom row (**C**,**D**) for Caco-2 cells. The light gray bars in (**A**,**C**) represent the amino acid peak area ratios of FPA/l-phenylalanine. The dark gray bars in (**A**,**C**) show the FPA/l-serine ratios, while the hatched bars indicate 1000 × FPA/l-glycine. The light gray bars in (**B**,**D**) indicate the l-phenylalanine/l-serine ratios. In (**B)**, the dark gray bars indicate the l-phenylalanine/l-glycine ratios, while in (**D**) the dark gray bars indicate 100 × l-phenylalanine/l-glycine ratios. The averages of five replicate samples were plotted (*n* = 5) and error bars denote ± standard deviation from the mean. An asterisk (*) indicates a significant difference from control samples (*p* ≤ 0.05) and a dagger (†) indicates a significant difference from the previous (lower) concentration (*p* ≤ 0.05).

As for m-tyr, there is a clear and steady increase in the FPA/l-phenylalanine ratios with increasing concentration of FPA as shown in Figure 8. Again, there is also a trend of increasing FPA/l-serine and FPA/l-glycine concentrations with increasing FPA exposure concentrations, as well as decreasing l-phenylalanine/l-serine and l-phenylalanine/l-glycine ratios as more l-phenylalanine is replaced with higher concentrations of FPA.

Both m-tyr and FPA yielded expected results for the amino acid compositional analysis, showing clear misincorporation. No BMAA was detected in any of the protein extracts from BMAA-exposed cells, at any of the exposure concentrations. Free, non-protein-associated BMAA was detected in the cell lysates of all exposed cells, confirming BMAA uptake by these cells. The l-serine/l-glycine and l-serine/l-phenylalanine ratios also did not change in BMAA-exposed cell protein hydrolysates. The changes observed in l-phenylalanine/l-serine and l-phenylalanine/l-glycine observed in m-tyr and FPA exposed cells, an indication of misincorporation of these analogues, was therefore absent in BMAA exposed cells. These data, in conjunction with the differences in cell viability and apoptosis/necrosis observed between known misincorporating analogues and BMAA, strongly suggest that BMAA does not misincorporate into proteins in these human cell lines, or that misincorporation occurs at a level that is undetectable and therefore toxicologically irrelevant. The absence of BMAA in protein extracts also confirms that the slight apoptosis and necrosis seen in BMAA-exposed HepG2 cells was not as a result of BMAA misincorporation, and suggests an additional mechanism of toxicity not related to excitotoxicity, such as the inhibition of enzymes reported by Esterhuizen-Londt et al. [76,77].

## 3. Conclusions

BMAA did not produce concentration-dependent apoptosis or necrosis, or affect the reduction of MTT in any of the tested cell lines. This is in direct contrast to the results observed for known misincorporating analogues FPA and m-tyr in the same cell lines. No BMAA was detected in purified protein extracts of BMAA-exposed cells, in direct contrast to the FPA and m-tyr detected in purified protein extracts from cells exposed to these analogues. No reduction in the encoded amino acid hypothesized to be replaced by BMAA was seen in any exposed cells, in direct contrast to the reduction in l-phenylalanine relative to other amino acids that was observed in m-tyr and FPA exposed cells. These data add to the growing body of evidence that BMAA is not misincorporated into proteins and that this therefore does not constitute a mechanism of toxicity.

## 4. Materials and Methods

### 4.1. Cell Culture

HepG2 cells, a human hepatocellular carcinoma cell line (Highveld Biological, Johannesburg, RSA), were routinely maintained in MEM/EBSS (Hyclone, GE Healthcare Bio-Sciences, Pittsburgh, PA, USA), with 2 mM l-glutamine, supplemented with 10% fetal bovine serum (FBS) (Hyclone) and penicillin–streptomycin (100 μ·mL^−1^). HeLa cells, a human cervical epithelial adenocarcinoma cell line (Cellonex, Johannesburg, RSA), and Caco-2 cells, a human colorectal epithelial adenocarcinoma (Highveld Biological, Johannesburg, RSA), were routinely maintained in RPMI-1640 (Hyclone), with 2.05 mM l-glutamine, supplemented with 10% FBS and penicillin–streptomycin. The cells were incubated in a humidified 95% oxygen/5% carbon dioxide incubator at 37 °C.

### 4.2. Metabolic Activity (MTT) Assay

HepG2, HeLa, and Caco-2 cells were seeded in 96-well plates at a density of 6.0 × 10^4^ cells·well^−1^ in 200 µL culture medium and allowed to attach overnight. Following attachment, culture media were replaced with 200 µL of treatment media. BMAA (Sigma Aldrich, St. Louis, MO, USA) from a 100 mM stock in 20 mM hydrochloric acid (HCl), l-m-tyrosine (Alfa Aesar, Haverhill, MA, USA) from a 500 mM stock in 1 M HCl and l-4-fluorophenylalanine (FPA) (Fluorochem, Derbyshire, UK) from a 270 mM stock in 0.5 M HCl, respectively, were added to the cells in concentrations ranging from 0.25 mM to 2 mM in the appropriate culture medium. Vehicles were used as controls. Treatments were continued for 48 h. Following treatments, the media were aspirated and replaced with 200 µL of either MEM/EBSS or RPMI-1640, depending on the cell line, containing 0.5 mg·mL^−1^ of 3-(4,5-dimethylthiazol-2-yl)-2,5-diphenyl tetrazolium bromide (MTT). The cells were incubated with the MTT media for 3 h at 37 °C, followed by aspiration of the media and replacement with 200 µL of dimethyl sulfoxide to solubilize the formazan crystals. The absorbance was subsequently measured at 540 nm using a Biotek PowerWave XS microtiter plate reader (Biotek, Winooski, VT, USA).

### 4.3. Phosphatidylserine Translocation (Apoptosis/Necrosis)

HepG2 and Caco-2 cells were seeded in 96-well plates at a density of 6.0 × 10^4^ cells·well^−1^ in 200 µL culture medium and allowed to attach overnight. The spent medium was subsequently aspirated from each well and replaced with treatment medium. Amino acid treatments were prepared in the same way as described in Section 2.2 with concentrations ranging from 0.5 to 2 mM. Vehicles were used as controls. After 24 h and 48 h of treatment, cell numbers and apoptosis/necrosis were determined using Hoechst staining in combination with an Annexin V-FITC Kit (Miltenyi Biotec, Singapore, Singapore). Briefly, treatment media were aspirated from the wells and replaced with 100 µL binding buffer, diluted with phosphate-buffered saline (PBS) as per kit instructions, containing Hoechst (1 µL·mL^−1^) and Annexin (10 µL·mL^−1^). The plates were incubated in the dark at room temperature for 15 min. Propidium iodide was subsequently added in 50 µL binding buffer to each well to a final concentration of 2 µg·mL^−1^ before analyzing the cells using the ImageXpress Micro XLS (Molecular Devices).

### 4.4. Exposure of Cells to Amino Acids and Preparation of Proteins for Analysis

HeLa, HepG2 and Caco-2 cells were seeded in 12-well plates at a density of 1.5 × 10^5^ cells·well^−1^ in a total of 2 mL of culture medium specific to each cell line. The cells were left to attach overnight. After attachment, culture media were removed from the cells and treatment media were added. The treatments were BMAA, m-tyr, and FPA, and these were prepared from stocks as described in Section 2.2 in the appropriate culture medium. Vehicles were used as controls. The treatments were continued for 24 h. Following treatments, the media were aspirated and the cells washed three times with PBS. The cells were subsequently collected by trypsinization and centrifugation in microcentrifuge tubes. Proteins were subsequently extracted from the cell pellets by trichloroacetic acid (TCA) extraction. Briefly, 100 µL of 20% TCA (in deionized water) were added to each cell pellet and left to stand on ice for 30 min. This was followed by 5 min of sonication in a 4 °C water bath sonicator. The samples were subsequently centrifuged at 20,800× *g* for 5 min at 4 °C to collect precipitated proteins. The supernatant, containing free amino acids, of each sample was collected. The protein pellets were subsequently washed twice more with 100 µL of 20% TCA to ensure that all the free amino acids were removed and the TCA extracts for each sample were pooled. The TCA extracts were subsequently dried using a Savant Speedvac. The protein pellets were washed with ice-cold acetone to remove residual TCA and left to dry. The protein pellets were further purified using sodium dodecyl sulfate polyacrylamide gel electrophoresis (SDS-PAGE) as per [78]. Briefly, 10% Tris-Glycine resolving gels with 6% stacking layers were prepared using the Bio-Rad Mini-PROTEAN^®^ Tetra Cell system with Bio-Rad buffers and reagents. Protein pellets were resuspended in 1 × loading buffer (0.0625 M Tris-HCl pH 6.8, 10% glycerol, 2% SDS, 1% β-mercaptoethanol, 0.01% bromophenol blue) heated to 95 °C for 5 min and loaded onto the gels. A voltage of 110 V was applied to the gels and the proteins were allowed to migrate until the dye front migrated roughly 2 cm into the resolving gel. The protein band for each sample was excised from the start of the resolving gel up to the dye front and the excised gel pieces placed in 0.22 µM Spin-X tubes (Corning^®^ Costar^®^). The gel pieces were broken up into pieces roughly 1 mm × 1 mm in size. Proteins were subsequently extracted from the gel pieces by addition of 0.25 M Tris-HCl pH 6.8 buffer with 0.1% SDS. The gel pieces were left in the solution overnight at 4 °C, and the supernatant for each sample was subsequently collected by centrifugation at 20,800× *g* at 4 °C for 15 min. A second aliquot of the extraction buffer was added to the gel pieces and left to stand for an hour before the supernatants were collected by centrifugation and pooled with the first aliquot. The supernatants were subsequently dried using a Speedvac. These dried protein extracts were subsequently acid hydrolyzed by adding of 100 µL of 6 N HCl to each sample in glass vials and incubating at 110 °C for 16 h. The hydrolysates were filtered through 0.22 µM Spin-X tubes before drying the samples using a Speedvac.

### 4.5. HPLC/MS-MS Analysis of Amino Acids

Dried protein hydrolysates were resuspended in 20 mM HCl and appropriately diluted for derivatization with 6-aminoquinolyl-*N*-hydroxysuccinimidyl carbamate (AQC) (Ultra AccQ-Tag derivatization kit for amino acid analysis. Waters, Milford, MA, USA). Labeled BMAA (l-BMAA-4,4,4-d_3_,^15^N_2_) (donated by the Institute of Ethnomedicine, at a purity of 99%) was added prior to derivatization as an internal standard. Derivatized samples were immediately mixed by vortexing and subsequently incubated at 55 °C for 10 min before analysis using a Shimadzu LC-20AB HPLC system coupled to a AB Sciex 4000 QTRAP^®^ hybrid triple quadrupole ion trap mass spectrometer. Sample separation was achieved by injecting 10 µL of the derivatized sample onto a Higgins (Mountain View, CA, USA) CLIPEUS^®^ C18 (5 µm, 15 cm × 3.0 mm) analytical column held at 55 °C, with gradient elution (0–0.5 min, 10% B; 6.0 min, 60% B; 7.0 min, 95% B; 11.0 min, 95% B; 11.50 min, 10% B; 15.0 min, 10% B), with mobile phases; water with 0.3% glacial acetic acid as solvent A and acetonitrile with 0.3% glacial acetic acid as solvent B, at a flow rate of 0.350 mL·min^−1^. The mass spectrometric analysis of the derivatized amino acid samples was achieved using a TurboIonspray^®^ source in positive ion detection mode. The parameters were as follows: a source temperature of 400 °C was used, ion spray voltage was set to 5500 V, ion source gas (nitrogen) 1 and 2 was set to 30 psi, and the curtain gas (also nitrogen) was set to 25 psi. A multiple reaction monitoring (MRM) scan type was used to measure the two most intense transitions of each of the amino acids and a dwell time of 40 ms was used for all transitions. The optimized collision energies and ion source potentials for each MRM transition are listed in Table 1. BMAA in protein hydrolysates was identified by retention time, parent ion mass, and specific and unique transitions for the analyte. The limits of BMAA detection (LoD) and quantification (LoQ) in the protein extract matrix were determined by adding isotopically labeled BMAA to appropriately diluted hydrolyzed protein extracts prior to derivatization. A peak with a signal to noise ratio (S/N) of at least 10 was selected as the LoD, while the S/N for the LoQ was 25. The LoD and LoQ were estimated to be 0.1 pg and 1 pg on column, respectively.

### 4.6. Statistical Analysis

Statistical significance of the data was determined using the non-parametric Mann–Whitney *U* test (α = 0.05).

## Figures and Tables

**Table 1:** The quantifier (Q) and qualifier (q) MRM transitions of all the target amino acids with the optimized declustering potential (DP), collision energy (CE) and collision cell exit potential (CXP) for each, are listed below.

Analyte	Q1 Mass (m/z)	Q3 Mass (m/z)	DP (V)	CE (V)	CXP (V)
l-glycine Q	246.14	171.00	61	29	14
l-glycine q	264.14	116.20	61	63	18
l-serine Q	276.17	170.90	71	37	28
l-serine q	276.17	115.90	71	71	18
l-phenylalanine Q	336.15	171.00	76	33	14
l-phenylalanine q	336.15	115.90	76	91	18
l-m-tyrosine Q	352.24	171.00	81	33	14
l-m-tyrosine q	352.24	116.00	81	91	18
l-4-fluorophenylalanine Q	354.16	171.00	66	33	14
l-4-flurophenylalanine q	354.16	116.00	66	89	18
l-BMAA Q	459.20	171.00	86	53	14
l-BMAA q1	459.20	119.00	86	31	10
l-BMAA q2	459.20	258.00	86	33	22
l-BMAA-4,4,4-d_3_,^15^N_2_ Q	464.21	171.00	86	53	1
l-BMAA-4,4,4-d_3_,^15^N_2_ q1	464.21	124.00	86	31	10
l-BMAA-4,4,4-d_3_,^15^N_2_ q2	464.21	259.20	86	33	22
